# Mechanistic Insights into Drug-Induced Guillain–Barré Syndrome: A Large-Cohort Analysis of the FAERS Database

**DOI:** 10.3390/ph18040498

**Published:** 2025-03-29

**Authors:** Jianxiong Gui, Xiao Li, Hongyuan Chu, Junjiao Zhang, Meiyu Dong, Fan Zhang, Renqiuguo Li, Huaxia Luo, Kai Gao, Yuwu Jiang

**Affiliations:** 1 Children’s Medical Center, Peking University First Hospital, No.5 Leyuan Road, Daxing District, Beijing 102627, China; gui199508046@163.com (J.G.); 2011110235@bjmu.edu.cn (X.L.); chuhongyuan@pku.edu.cn (H.C.); docter_zzhang@163.com (J.Z.); dongmeiyu7980@163.com (M.D.); cmufanzhang@163.com (F.Z.); rl4940@nyu.edu (R.L.); huaxia.luo@pkufh.com (H.L.); gaokaipku@bjmu.edu.cn (K.G.); 2 Beijing Key Laboratory of Molecular Diagnosis and Study on Pediatric Genetic Diseases, Beijing 102627, China; 3 Children Epilepsy Center, Peking University First Hospital, Beijing 102627, China; 4 Key Laboratory for Neuroscience, Ministry of Education/National Health and Family Planning Commission, Peking University, Beijing 102627, China; 5 Center of Epilepsy, Beijing Institute for Brain Disorders, Beijing 102627, China

**Keywords:** Guillain–Barré Syndrome, Food and Drug Administration Adverse Event Reporting System, Mendelian Randomization, adverse drug reactions, pharmacovigilance

## Abstract

**Background/Objectives**: Drug-induced Guillain–Barré Syndrome (GBS) is a severe complication of pharmacotherapy. Previous research has established a connection between certain medications and higher GBS risk. However, a large-cohort analysis is crucial to reveal underlying biological mechanisms of drug-induced GBS. This study aimed to evaluate the association between GBS and various drugs currently accessible in the Food and Drug Administration Adverse Event Reporting System (FAERS) database and explore the mechanisms underlying drug-induced GBS. **Methods**: We analyzed drug-induced GBS adverse event reports in the FAERS database to identify strongly associated drugs. We then investigated GBS susceptibility proteins through GWAS meta-analysis and Mendelian Randomization (MR) based on plasma proteomics, complemented by protein–protein interaction (PPI) network analysis to explore underlying mechanisms. **Results**: A total of 4094 FAERS reports were analyzed, leading to the selection of 30 drugs with the highest signal strength and 54 drug targets. MR analysis identified 73 susceptibility proteins linked to GBS risk. PPI analysis revealed that 10 genes encoding GBS-susceptible proteins were associated with 19 drug target genes involved in 13 different drugs. Among these, the antineoplastic drug Nelarabine showed the strongest correlation with GBS. The *TNF* and *PDCD1LG2* genes emerged as key GBS-susceptible genes. Additionally, *TNF* was negatively correlated with GBS, and *PDCD1LG2* was positively correlated with GBS. KEGG analysis indicated that pyrimidine metabolism, purine metabolism, and the IL6/JAK/STAT3 signaling pathway also significantly contribute to drug-induced GBS. **Conclusions**: This study improved our understanding of the biological mechanisms of drug-induced GBS, thereby pinpointing potential therapeutic targets for future intervention.

## 1. Introduction

Guillain–Barré Syndrome (GBS) is an acute inflammatory polyradiculoneuropathy affecting the peripheral nervous system, marked by progressive limb weakness, sensory deficits, cranial nerve involvement, and cerebrospinal fluid (CSF) albuminocytological dissociation. It is the leading cause of acute flaccid paralysis globally [1]. The worldwide incidence of GBS ranges from 0.16 to 3.0 cases per 100,000 person-years [2,3]. Drug-induced GBS is a life-threatening condition initially identified in individuals who received the influenza vaccine [4]. Associated drugs include allopurinol, gold therapy, gangliosides, TNF-alpha antagonists, second-generation antipsychotics like risperidone, and some antibiotics [5,6,7,8]. Most reports on drug-induced GBS are sporadic, with only a few medications occasionally documented in literature reviews and summaries regarding their potential to trigger GBS. For instance, it has been reported that the frequency of demyelinating diseases in patients receiving anti-TNF-α therapy ranges from approximately 0.02% to 0.2% [9]. Moreover, a study on neuroimmunological adverse events associated with immune checkpoint inhibitors (ICIs) reported that the proportion of GBS in patients treated with ICIs was nearly eight times higher than in the control group [10]. GBS with potentially life-threatening consequences occurs in approximately 0.1–0.2% of patients treated with ICIs [11].Currently, the specific details and underlying mechanisms are unclear, necessitating a comprehensive analysis of large patient samples to elucidate the characteristics of these commonly used drugs and mechanisms in drug-induced GBS.

The Food and Drug Administration Adverse Event Reporting System (FAERS) is a voluntary system that collects information on drug-related adverse events reported by healthcare professionals, patients, and manufacturers. It provides valuable insights into drug safety and serves as an essential resource for monitoring adverse reactions to pharmaceuticals [12]. However, this database has not yet been fully utilized to investigate drug-induced GBS. To explore the underlying mechanisms and gain a comprehensive understanding of drug-induced GBS, we analyzed all reports of drug-induced GBS currently available in the FAERS database. Mendelian Randomization (MR) and genome-wide association study (GWAS) meta-analysis based on plasma proteomics were employed for drug target identification and the identification of GBS susceptibility proteins. To further investigate the association between drug use and GBS, protein–protein interaction (PPI) network analysis was also utilized. By integrating GWAS, MR, and PPI network analysis, this study aimed to improve our understanding of how specific drugs may trigger this serious condition.

## 2. Results

**Figure 1:**
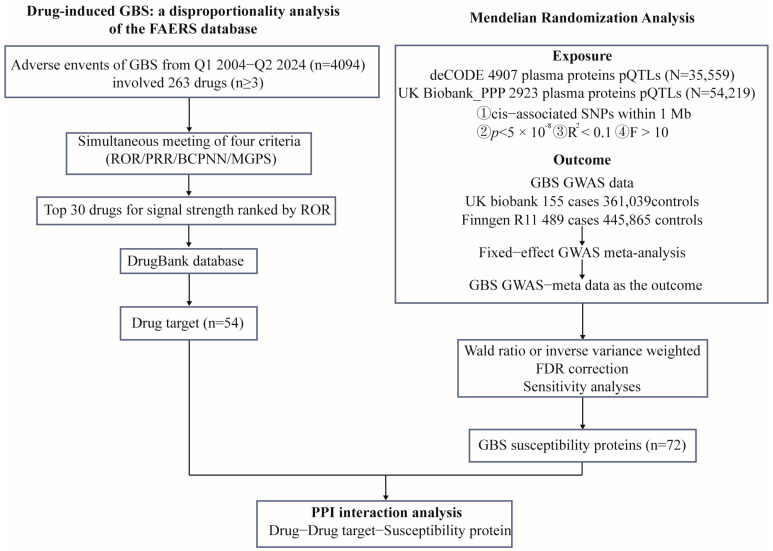
Instrumental variables selection and analysis process flow chart. MR, Mendelian Randomization; FAERS, FDA Adverse Event Reporting System; GBS, Guillain–Barre Syndrome.

The schematic presentation of the overall study design is shown in Figure 1.

### 2.1. Descriptive Analysis

Clinical characteristics of reports with drug-induced GBS from the FAERS database (2004 Q1–2024 Q2).

After the removal of duplicates and subsequent filtering, a total of 4094 reports of drug-induced GBS were identified. The clinical characteristics associated with these reports from the FAERS database were presented in Table 1. Notably, the proportion of males (49.98%) was significantly higher than that of females (38.86%). Regarding age distribution, although the ages of 27.58% of patients remained unknown, most reported cases occurred within the middle-aged and elderly demographic (41–64 years old), which accounted for 31.31%. The incidence rate of drug-induced GBS among pediatric patients (≤18 years old) was 3.86%. Among all reported data, the United States represented the largest share at 24.94%. In terms of clear indications, most patients (82.49%) had well-documented records indicating specific drugs responsible for causing GBS.

**Figure 2:**
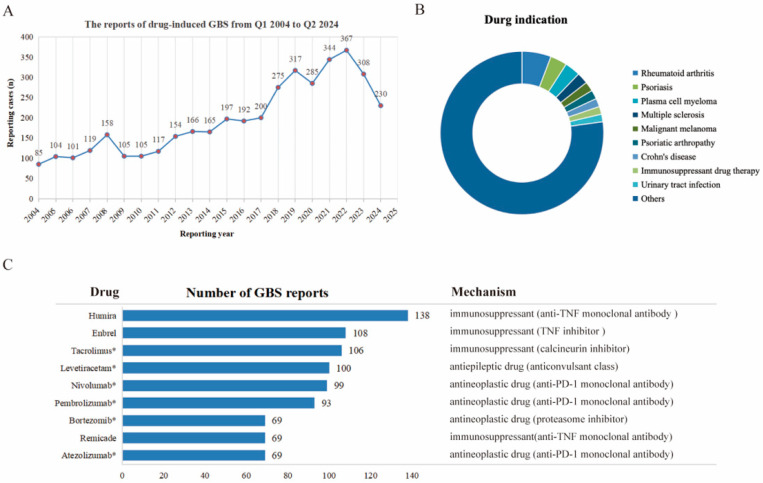
Characteristics of drugs inducing GBS and possible drug targets. (**A**) Number of annual reports of drug-induced GBS from Q1 2004 to Q2 2024; (**B**) an overview of the typical medical conditions for which drugs associated with GBS induction were prescribed, highlighting the clinical contexts in which these drugs were commonly used; (**C**) a comparative ranking of drugs based on the number of reported cases of drug-induced GBS. * indicated a ROR > 3, highlighting drugs that exhibit significant signal strength.

Further analysis of drugs associated with GBS revealed their primary uses in treating conditions such as rheumatoid arthritis, psoriasis, plasma cell myeloma, multiple sclerosis, malignant melanoma, psoriatic arthropathy, Crohn’s disease, immunosuppressant therapy, and urinary tract infections (Figure 2B). Figure 2C presented the drugs with the highest number of reported cases, ranked as follows: Humira (n = 138), Enbrel (n = 108), Tacrolimus (n = 106), Levetiracetam (n = 100), Nivolumab (n = 99), Pembrolizumab (n = 93), Bortezomib (n = 69), Remicade (n = 69), and Atezolizumab (n = 69). Apart from Levetiracetam, all the listed drugs are classified as either immunosuppressants or antineoplastic agents.

### 2.2. Signal Strength Detection

If we consider only the number of cases, it would not provide a comprehensive view. A higher number of reports linking a particular drug to drug-induced GBS might simply reflect its widespread use, rather than indicating the strongest association. Therefore, we employed four well-established drug association analysis formulas to identify the drugs with the strongest correlation to GBS. Table 2 presents the top 30 drugs with the highest signal strength, ranked by ROR. The top five drugs were Nelarabine (n = 16, ROR 138.91, PRR 134.71, IC 7.07, EBGM 134.19), Roferon-a (n = 3, ROR 60.62, PRR 59.81, IC 5.90, EBGM 59.77), Zerit (n = 3, ROR 30.17, PRR 29.97, IC 4.90, EBGM 29.95), Methimazole (n = 9, ROR 22.43, PRR 22.32, IC 4.48, EBGM 22.28), and Pravastatin sodium (n = 4, ROR 22.25; PRR 22.15; IC 4.47; EBGM 22.13).

**Figure 3:**
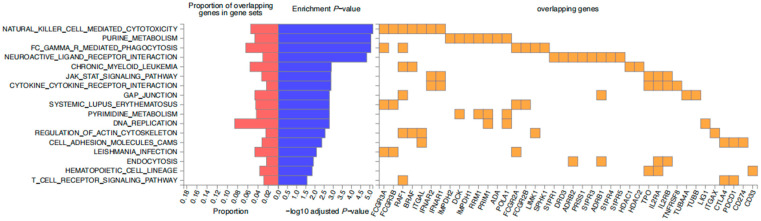
Analysis of genes recoding drug targets and their potential signaling pathways. Listed on the left side are the names of different gene enrichment pathways, such as “Natural Killer Cell Mediated Cytotoxicity”, “Purine Metabolism”, etc.; in the middle part, the red bars indicates the proportion of overlapping genes in gene sets. The longer the red bar, the higher the proportion of overlapping genes. Blue bars represent *p*-values (Enrichment *p*-value), indicating significance differences; shown on the right side are overlapping genes that encode drug targets (overlapping genes), represented by orange squares.

Moreover, the drug targets and mechanism for these medications are presented in Supplementary Table S1, based on the Drug Bank database. To further elucidate the relevance between the drug targets of medicines inducing GBS, KEGG (Kyoto Encyclopedia of Genes and Genomes) pathway analysis was performed. This analysis identified several pivotal pathways, with the top five being natural killer cell-mediated cytotoxicity, purine metabolism, Fc gamma R-mediated phagocytosis, neuroactive ligand-receptor interaction, and the chronic myeloid leukemia signaling pathway (Figure 3).

### 2.3. Potential Susceptibility Proteins for GBS

**Figure 4:**
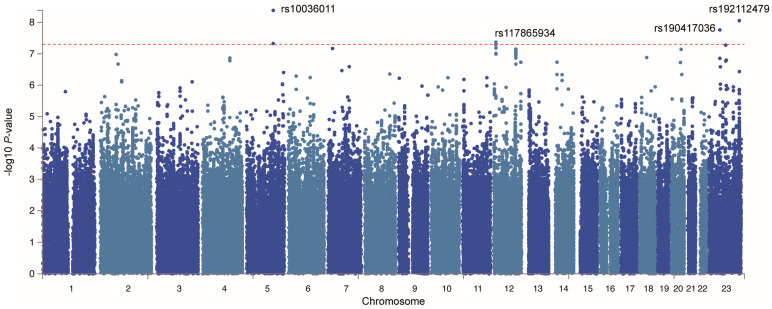
The Manhattan plot of GWAS meta-analysis for significant SNP loci. Revealing four significant SNP loci with *p*-values less than 5 × 10^−8^.

To identify potential GBS susceptibility proteins, we first conducted a GWAS meta-analysis by integrating GBS GWAS data. The QQ plot derived from the GWAS meta-analysis is presented in Figure S1, revealing a genomic inflation factor (λ) of 1.015. Typically, a value greater than 1 indicates acceptable stability, providing reliable outcome data for subsequent MR analyses. Our investigation revealed four significant SNP loci (Figure 4, Table S2) with *p*-values less than 5 × 10^−8^, as depicted in the Manhattan Plot of GWAS meta-analysis, suggesting a statistically significant association with a specific trait or disease. However, as shown in the Manhattan plot based on genetic analysis, we were unable to directly identify genetic risk loci significantly associated with GBS (*p* < 5 × 10^−8^), which could be attributed to the relatively small sample size of GBS cases (Figure S2).

**Figure 5:**
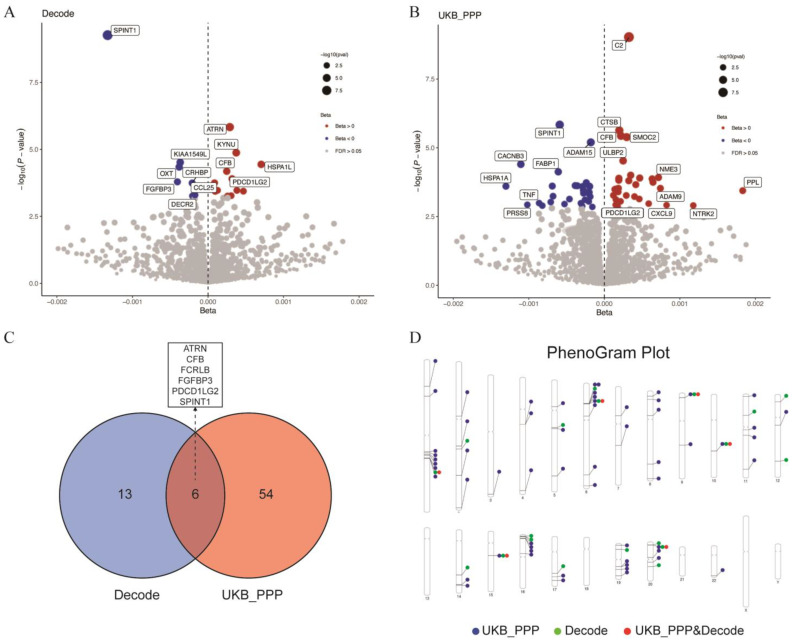
Result summary of MR and colocalization analysis on the associations between plasma proteins and the risk of GBS. (**A**,**B**) The scatter plots in Chart A (Decode) and Chart B (UKB_PPP) illustrate the relationship between proteins and their respective −log_10_ (*p*-value) and Beta values. The points were color-coded to represent different Beta value ranges: red for Beta > 0, blue for Beta < 0, and gray for FDR > 0.05. Each point corresponded to a protein, with its position on the chart reflecting its −log_10_ (*p*-value) and Beta value; (**C**) this Venn diagram illustrates the overlap of proteins between the Decode and UKB_PPP datasets. The blue circle on the left denotes the number of proteins in the Decode dataset (13), while the orange circle on the right indicates the number of proteins in the UKB_PPP dataset (54). The overlapping section in the center represents the number of genes shared by both datasets (6); (**D**) the chromosomal distribution of these genes encoding 73 proteins. Full name of proteins: ATRN, attractin; CFB, complement factor B; FCRLB, Fc receptor-like B; FGFBP3, fibroblast growth factor binding protein 3; PDCD1LG2, programmed cell death 1 ligand 2; SPINT1, serine peptidase inhibitor, Kunitz type 1; KYNU, kynureninase; KIAA1549L, KIAA1549 like; OXT, oxytocin/neurophysin I prepropeptide; CRHBP, corticotropin-releasing hormone binding protein; HSPA1L, heat shock protein family A (Hsp70) member 1 like; CCL25, C-C motif chemokine ligand 25; DECR2, 2,4-dienoyl-CoA reductase 2; C2, complement C2; CTSB, cathepsin B; SMOC2, SPARC related modular calcium binding 2; ADAM15, ADAM metallopeptidase domain 15; ULBP2, UL16 binding protein 2; CACNB3, calcium voltage-gated channel auxiliary subunit beta 3; FABP1, fatty acid binding protein 1; TNF, tumor necrosis factor; PRSS8, serine protease 8; NME3, NME/NM23 nucleoside diphosphate kinase 3; ADAM9, ADAM metallopeptidase domain 9; CXCL9, C-X-C motif chemokine ligand 9; NTRK2, neurotrophic receptor tyrosine kinase 2.

Subsequently, MR analysis was conducted using plasma proteomic data as exposure and GBS GWAS-meta data as the outcome. In the Decode dataset, 20 proteins associated with GBS risk were identified (Table S3a), while 61 proteins were identified in the UKB_PPP dataset (Table S3b). Following sensitivity and heterogeneity analyses, a total of 73 GBS susceptibility proteins were included, comprising 19 from Decode and 60 from UKB_PPP (Figure 5A,B), with six proteins shared between the two datasets: attractin (ATRN), complement factor B (CFB), Fc receptor-like B (FCRLB), fibroblast growth factor binding protein 3 (FGFBP3), programmed cell death 1 ligand 2 (PDCD1LG2), and serine peptidase inhibitor, Kunitz type 1 (SPINT1) (Figure 5C). Reverse MR analysis, which used GBS GWAS-meta data as the exposure and plasma proteomic data as the outcome, suggested no reverse causal relationship between GBS and the 73 proteins (Table S3c). This finding suggested that the potential reverse causal effects of GBS on these proteins had been thoroughly considered and excluded. Consequently, this reinforced the hypothesis that these proteins were likely involved in susceptibility to GBS. The chromosomal distribution of these genes encoding 73 proteins is illustrated in Figure 5D.

### 2.4. PPI Network and Enrichment Analysis Results

**Figure 6:**
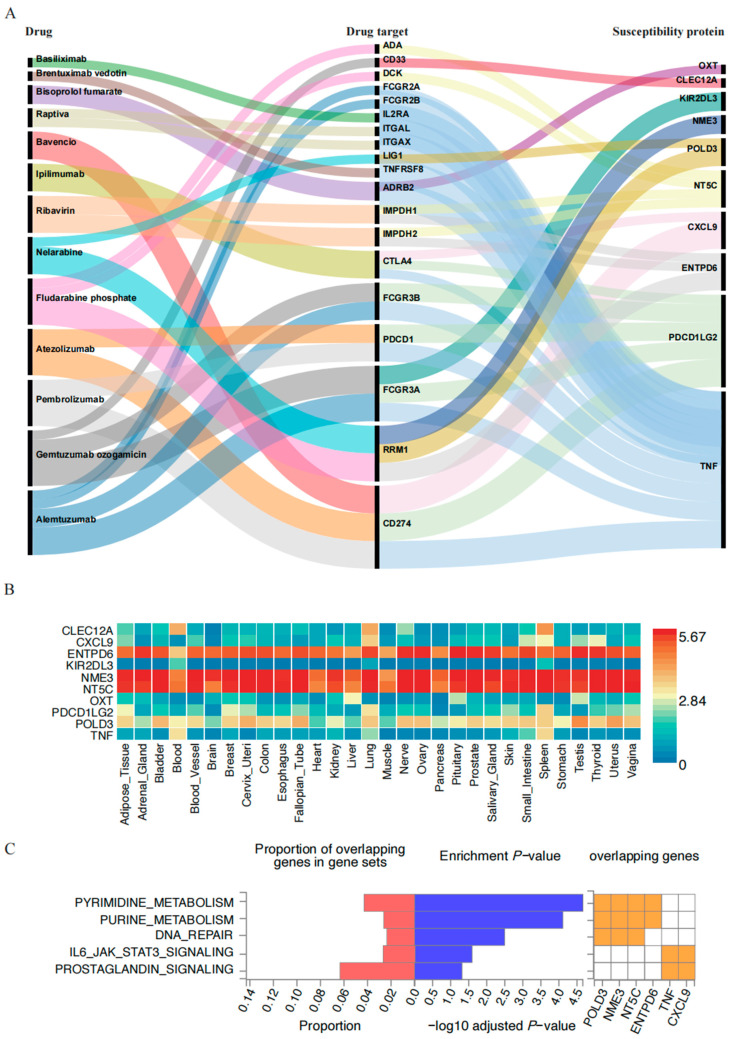
Result summary of associations between GBS-related proteins and drug target of GBS-induced drugs. (**A**) Protein–protein interaction network of the GBS susceptibility proteins and drug targets; (**B**) the expression levels of specific proteins across various tissues; (**C**) analysis of GBS susceptibility genes and their potential signaling pathways.

Additionally, we generated PPI networks to explore the interactions between the 73 GBS susceptibility proteins and previously identified drug targets (Figure S3, Table S4). The PPI network provided a detailed visualization of the potential interactions between drugs and their putative targets, as well as the connections between genes encoding GBS-related proteins and these drug targets. This comprehensive illustration enhanced our understanding of the intricate relationships within the network, highlighting potential avenues for therapeutic intervention. A total of 10 genes (out of 73) encoding GBS-susceptible proteins were found to interact with 19 genes (out of 54) encoding drug targets that were potentially associated with GBS. These 19 drug targets were involved in a total of 13 different drugs (Figure 6A). The top five GBS susceptibility genes with the most association with drug targets were the following: Tumor Necrosis Factor (TNF, 15 interactions), Integrin Subunit Alpha X (ITGAX, nine interactions), Fc gamma receptor IIIa (FCGR3A, six interactions), 5′, 3′-nucleotidase, cytosolic (NT5C, five interactions), and PDCD1LG2 (five interactions). By analyzing PPI networks, we identified key genes associated with drug targets. The GBS susceptibility-related gene TNF had the highest number of interactions, with 15 drug targets. The drugs associated with these targets included Bisoprolol fumarate (cardiovascular drug), Atezolizumab (antineoplastic drug), Alemtuzumab (immunosuppressant), Bavencio (antineoplastic drug), Pembrolizumab (antineoplastic drug), Ipilimumab (antineoplastic drug), Basiliximab (immunosuppressant), and Raptiva (immunosuppressant), with Alemtuzumab (immunosuppressant) being the most closely related. PDCD1LG2 was the second most interactive protein, binding to five drug targets, with Gemtuzumab ozogamicin (antineoplastic drug) being the most closely associated drug.

Subsequently, we performed an organ system distribution analysis of these 10 GBS-related proteins. Figure 6B illustrated the expression levels of specific proteins in different tissues, aiding in understanding their functions and distributions across various tissues. Notably, TNF, DNA polymerase delta 3 (POLD3), PDCD1LG2, Killer cell immunoglobulin like receptor, two Ig domains and long cytoplasmic tail 3 (KIR2DL3), Ectonucleoside triphosphate diphosphohydrolase 6 (ENTPD6), C-X-C motif chemokine ligand 9 (CXCL9), and C-type lectin domain family 12 member A (CLEC12A) exhibited high expression in immune-related tissues (such as blood and spleen), suggesting their potential involvement in immune responses and inflammatory processes.

To explore the possible biological mechanisms of GBS-related genes identified by MR, we conducted enrichment analysis on these 10 genes, filtering the results to include only pathways with a corrected *p* < 0.05 for significance. We identified a total of five pathways associated with GBS (Figure 6C). These genes were primarily involved in the following processes: pyrimidine metabolism, purine metabolism, DNA repair, IL6/JAK/STAT3 signaling, and prostaglandin signaling.

## 3. Discussion

GBS is an immune-mediated polyneuropathy characterized by areflexia and ascending paresthesia, which can progress to respiratory failure [1]. The use of certain drugs, particularly some ICIs employed in immunotherapy for melanoma and refractory cancers, may elevate the risk of developing GBS [11,13]. However, the possible mechanisms and triggers of GBS in patients receiving specific medications remain unknown. This study focused on occurrences of GBS associated with drugs use as recorded in the FAERS database and explored potential mechanisms through MR analysis and PPI analysis.

In this study, the number of reported cases of drug-induced GBS has increased steadily from 2019 to 2022, reaching its peak in 2022. Recent reports found that the COVID-19 virus and its vaccines can increase the incidence of GBS by two to five times, leading to more severe cases of GBS in patients [14,15]. Furthermore, the first report of GBS caused by COVID-19 and its vaccines were in 2019 and 2021, respectively [15,16]. Therefore, we speculate that the increased incidence of drug-induced GBS during this period may be related to the immune system disorders caused by COVID-19 and its vaccines [16].

Our findings indicated that drug-induced GBS was predominantly associated with medications utilized in the treatment of immune system disorders and cancers. Among the 13 drugs associated with the 10 GBS-susceptible genes, eight were antineoplastic drugs, three were immunosuppressant drugs, one was a cardiovascular drug, and one was an antiviral drug. Among these antineoplastic drugs, Nelarabine exhibited the strongest correlation with GBS. Nelarabine is a purine nucleoside analog used in the management of T-cell acute lymphoblastic leukemia (T-ALL) and T-cell lymphoblastic lymphoma (T-LBL) [17]. Its primary mechanism involves disrupting DNA synthesis by inhibiting Ribonucleotide Reductase M1 (RRM1), thereby hindering the proliferation of cancer cells. Additionally, Nelarabine may induce apoptosis in cells with compromised LIG1 function [18]. This study validated that the drug targets of Nelarabine were RRM1 and LIG1 (DNA ligase 1); however, the mechanisms underlying its side effects that lead to GBS remain unclear. PPI analysis in this study suggested that Nelarabine-induced GBS may be mediated via the RRM1/LIG1-POLD3 or RRM1-NME3 pathway. POLD3 is essential for DNA repair and maintaining immune cell function [19]. Previous studies have indicated that LIG1 level can influence the expression of POLD3 [20,21]. Additionally, NME3 (Nucleoside Metabolism Enzymes 3) plays a significant role in repairing both single- and double-stranded breaks in DNA, contributing to genomic instability and promoting malignant tumor progression [22,23]. This study proposed that the drug target RRM1 may induce GBS through POLD3 and NME3 proteins. However, the biological significance of the results requires further experimental verification.

The three immunosuppressant drugs Raptiva, Basiliximab and Alemtuzumab were all linked to the *TNF* gene, which opens new avenues for understanding and treating this debilitating condition. These medications are widely utilized in the treatment of various autoimmune and inflammatory diseases. Several studies indicate that TNF-α produced by T cells plays a crucial pro-inflammatory role in GBS, contributing to demyelination and axonal damage in peripheral nerves [24]. In 1993, one study documented that elevated serum concentrations of TNF-α are detectable in 20% to 50% of patients diagnosed with GBS [25]. The release of TNF-α may exacerbate inflammatory demyelination [26]. However, the MR analysis in Figure 6B shows a negative association between TNF expression and drug-induced GBS. Anti-TNF-α drugs have been linked to an increased incidence and activity of demyelinating diseases like GBS [27]. This paradox may be explained by the dual role of TNF-α: while it promotes inflammation, it also has immunoregulatory functions, suppressing T cell reactivity to autoantigens. Systemic anti-TNF-α therapy could penetrate the peripheral nervous system where the blood–nerve barrier is weak, potentially neutralizing local TNF-α and disrupting its balance [28]. Reduced TNF-α levels could prolong the activation of myelin-specific T cells, raising the risk of immune-mediated neuropathy in susceptible individuals [29]. These findings emphasized that patients undergoing anti-TNF-α therapy should be closely monitored for neurological symptoms indicative of GBS. Additionally, if patients experience GBS-related adverse reactions while taking medications potentially associated with TNF-α in Figure 6A, the possibility of persistently activated myelin-specific T cells due to excessively low TNF-α levels should be considered. Therefore, when evaluating and treating such patients, clinicians should pay attention to the immunomodulatory role of TNF-α and its impact on the nervous system to develop more precise treatment strategies.

Additionally, PDCD1LG2 (PD-L2) emerged as another significant protein that was positively associated with drug-induced GBS, as indicated in Figure 5A,B. The PPI analysis carried out in this study presented potential associations between PDCD1LG2 and the drug targets of Ipilimumab, Gemtuzumab ozogamicin, Atezolizumab, Pembrolizumab, Bavencio and Alemtuzumab. These drugs are all monoclonal antibodies used in cancer treatment [30,31,32,33]. However, there have been no previous reports linking GBS to PDCD1LG2 protein. PDCD1LG2 is an important immunoregulatory molecule primarily expressed in antigen-presenting cells (APCs) [34]. It regulates T cell activation and suppresses immune responses through its interaction with the receptor PD-1 [35]. This study suggests that caution is warranted regarding the occurrence of GBS when using the aforementioned monoclonal antibodies. PDCD1LG2 inhibitors may serve as a novel therapeutic target for preventing drug-induced GBS. Furthermore, investigating the mechanism of PDCD1LG2 in GBS could aid in enhancing the safety of immunotherapy.

Unexpectedly, the use of the cardiovascular drug Bisoprolol fumarate was also associated with the occurrence of GBS, a finding that had never been reported in the literature before. This study revealed that the β2 adrenergic receptor (ADRB2) was the drug target for Bisoprolol fumarate. ADRB2 is a G protein-coupled transmembrane receptor that is widely recognized as a pharmacological target for the treatment of asthma and chronic obstructive pulmonary disorder (COPD) [36]. Previous literature indicated that overactivation of ADRB2 can lead to a decrease in TNF-α levels [36]. Therefore, if neurological symptoms resembling GBS arise during the use of such medications, this mechanism could be taken into consideration.

To explore additional mechanisms, we conducted KEGG pathway analyses on genes encoding drug targets associated with GBS as well as 10 GBS susceptibility-related genes. The results revealed three common pathways: purine metabolism, IL6-JAK-STAT3 signaling, and pyrimidine metabolism. Previous studies suggest that metabolic factors may also play a role in the onset and progression of the disease [37,38]. Patients with metabolic syndrome exhibiting abnormalities in purine metabolism has been associated with an increased risk of developing GBS. Certain metabolic indicators (such as uric acid levels) in GBS patients may correlate with disease severity or prognosis [37,39]. Additionally, certain drugs that promote pyrimidine degradation may aid in GBS treatment [40]. To clarify this phenomenon, we conducted KEGG and PPI network analyses to identify GBS-related proteins involved in purine and pyrimidine metabolic pathways. We identified four such proteins: POLD3, NME3, NT5C, and ENTPD6. Through drug target analysis, these proteins were associated with three medications—Ribavirin, Nelarabine, and Fludarabine phosphate—all of which are purine or pyrimidine nucleoside analogs [41,42]. The potential mechanism underlying their action may involve the activation and function of immune cells being dependent on metabolic pathways, including purine and pyrimidine metabolism [43]. Metabolic dysregulation could impact immune cell activation and inflammatory responses. This finding offers new insights for clinical treatment strategies.

## 4. Materials and Methods

### 4.1. Data Sources and Processing

The data for this study were obtained from drug-induced GBS data in the FAERS database (https://open.fda.gov/data/faers/) (accessed on 28 September 2024) from Q1 2004 to Q2 2024. The reporting odds ratio (ROR) [44], proportional reporting ratio (PRR) [45], Bayesian confidence propagation neural network (BCPNN) [46], and multi-item gamma-Poisson shrinker (MGPS) [47] models were simultaneously applied to calculate signal values to yield more rigorous signals. Detailed criteria and specific calculations are listed in Table 3. The Medical Dictionary for Regulatory Activities (MedDRA) was used to identify drug-induced GBS cases, and the DrugBank database was used to determine drug generic names and drug targets. Additionally, the study explored the causal effect of gene variants linked to protein quantitative trait loci (pQTLs) on GBS risk using MR analysis. This study utilized gene variants linked to 4907 plasma proteins from the deCODE project, which included a sample size of 35,559 Icelanders [48], as well as gene variants related to 2923 plasma proteins from the UK Biobank_PPP project with a sample size of 54,219 participants [49], as exposure factors. Details on protein measurement, data processing, and quality control are available in the original studies.

We performed a fixed-effect GWAS meta-analysis using the METAL package as outcome factors [48,49], which integrated data from the UK Biobank (155 GBS cases and 361,039 controls) and FinnGen R11 (489 GBS cases and 445,865 controls). The quantile–quantile plot was generated using the ‘qqman’ package in R (version 4.3.0) [50]. Regional association plots for the lead SNP at each genome-wide significant locus were generated using the ‘gassocplot’ package in R (version 4.3.0) [51]. The results of the GWAS meta-analysis were annotated using FUMA (https://fuma.ctglab.nl/) (accessed on 4 October 2024) [52].

### 4.2. Mendelian Randomization

To fulfill the fundamental assumptions of MR—namely relevance, independence, and exclusion restriction—it is imperative to ensure the robustness of MR findings. The relevance assumption stipulates that IVs must exhibit a strong association with exposure. Independence necessitates that IVs are uncorrelated with confounding factors that could distort the exposure–outcome relationship. The exclusion restriction assumption asserts that IVs affect the outcome solely through their influence on the exposure, without any alternative pathways. The criteria for selecting pQTLs were as follows [53]: (1) cis-associated SNPs within 1 Mb of the gene encoding the target protein; (2) genome-wide significance defined by *p* < 5 × 10^−8^; (3) no significant linkage disequilibrium (LD), indicated by R^2^ < 0.1 in the 1000 Genomes European population; and (4) an F-statistic greater than 10 for each protein’s pQTL.

The MR analysis mainly employed both Wald ratio and inverse variance weighted (IVW) methods to evaluate the causal effect of plasma protein-related gene variants on GBS risk. Depending on the number of SNPs utilized as IVs, either IVW or the Wald ratio method was employed for each protein, using the ’TwoSampleMR’ package in R (version 4.3.0). To ensure the robustness of our findings, sensitivity analyses were conducted using MR-Egger regression, weighted median, weighted mode, and simple mode to detect directional pleiotropy [49]. Horizontal pleiotropy was evaluated through the MR-Egger intercept test, and heterogeneity was assessed using Cochran’s Q-test in both IVW and MR-Egger analyses. The presence of horizontal pleiotropy and heterogeneity can be ruled out if the following conditions are satisfied: the *p*-value of the MR-Egger intercept is >0.05, the *p*-value of Cochran’s Q test is >0.05, and the effect estimates of IVW are directionally consistent with at least two other estimation methods. Odds ratios (ORs) and 95% confidence intervals (CIs) were applied to quantify the associations between proteins and outcomes. A 5% false discovery rate (FDR) correction was applied to address multiple comparisons to avoid Type I errors in finding genetic associations [54]. In the reverse MR analysis, GBS was considered the exposure (with IVs meeting the criteria *p* < 5 × 10^−8^, R^2^ < 0.1, and F-statistic > 10), while plasma proteins were treated as the outcomes. Reverse causality estimates were similarly adjusted for multiple testing using FDR correction across all tested protein traits.

### 4.3. PPI Network and Enrichment Analysis

To investigate the potential mechanisms underlying drug-induced GBS, PPI analysis was conducted to assess the interactions between GBS susceptibility proteins and drug targets using the STRING database (https://string-db.org, accessed on 24 March 2025) [55]. The average expression levels of these critical protein-coding genes were examined across 54 human tissues using GTEx v8 data and presented as a heatmap. Furthermore, gene enrichment analysis revealed potential pathways associated with these genes using the ‘GENE2FUNC’ tool in FUMA (https://fuma.ctglab.nl/, accessed on 24 March 2025) [52].

### 4.4. Limitations

We conducted an in-depth investigation into the possible mechanisms of drug-induced GBS through FAERS, Mendelian Randomization, and Protein Interaction Networks, but there are still some limitations. Firstly, FAERS is based on voluntary reporting, which may introduce bias and result in underreporting of adverse events (AEs), thereby affecting the accuracy of the data. Secondly, the limited number of cases available in GWAS related to GBS restricted our ability to identify genes associated with GBS risk. Furthermore, both GWAS and proteomic datasets predominantly originated from populations of European ancestry; thus, caution should be exercised when extrapolating findings regarding GBS susceptibility proteins to other demographic groups. Our exploration of potential mechanisms between susceptible proteins and drug targets was based on bioinformatics predictions that require validation through biological experiments or clinical cohort studies in the future.

## 5. Conclusions

Our results investigated the association between drug use and GBS by analyzing adverse event reports from the FAERS and employing MR and PPI network analysis to explore underlying mechanisms. The study identified specific drugs strongly associated with GBS, such as Nelarabine. The findings suggested *TNF* and *PDCD1LG2* as potential therapeutic targets for drug-induced GBS, offering insights into both immune regulation and metabolic pathways involved in the disease. Our research provided possible biological mechanisms of drug-induced GBS, offering potential targets for therapeutic intervention and further research into the pathogenesis of GBS.

## Figures and Tables

**Table 1:** Clinical characteristics of reports with drug-induced GBS from the FAERS database (2004 Q1–2024 Q2).

Variable	Cases n (%)
**Sex**	
Female	1591 (38.86)
Male	2046 (49.98)
Unknown	457 (11.16)
**Age**	
≤18	158 (3.86)
19–40	503 (12.29)
41–64	1282 (31.31)
≥65	1022 (24.96)
Unknown	1129 (27.58)
**Reporter**	
Physician	1679 (41.01)
Consumer	792 (19.35)
Others	1429 (34.90)
Unknown	194 (4.74)
**Reported countries**	
United States	1021 (24.94)
France	266 (6.50)
United Kingdom	201 (4.91)
Others	1598 (39.03)
Unknown	1008 (24.62)
**Indication**	
Clear indication	3377 (82.49)
Unknown	717 (17.51)

**Table 2:** The top 30 drugs with the highest signal strength in the FAERS database were selected, based on the four algorithm standards.

Drug	Cases	ROR (95% CI)	PRR (95% CI)	*χ* ^2^	IC (IC-2SD)	EBGM (EBGM05)
Nelarabine	16	138.91 (84.37, 228.71)	134.71 (82.53, 219.89)	2115.68	7.07 (6.37)	134.19 (88.42)
Roferon-a	3	60.62 (19.39, 189.51)	59.81 (19.57, 182.8)	173.4	5.90 (4.47)	59.77 (23.03)
Zerit	3	30.17 (9.69, 93.96)	29.97 (9.62, 93.41)	83.98	4.90 (3.48)	29.95 (11.58)
Methimazole	9	22.43 (11.64, 43.21)	22.32 (11.69, 42.62)	182.94	4.48 (3.58)	22.28 (12.87)
Pravastatin sodium	4	22.25 (8.33, 59.47)	22.15 (8.31, 59.02)	80.71	4.47 (3.20)	22.13 (9.72)
Vinorelbine	3	22.10 (7.10, 68.75)	21.99 (7.06, 68.54)	60.09	4.46 (3.04)	21.98 (8.5)
Gemtuzumab ozogamicin	3	21.11 (6.79, 65.66)	21.02 (6.74, 65.51)	57.16	4.39 (2.97)	21.00 (8.13)
Raptiva	11	19.88 (10.99, 35.97)	19.8 (11.00, 35.65)	195.82	4.30 (3.48)	19.74 (12.02)
Brentuximab vedotin	29	19.72 (13.68, 28.44)	19.64 (13.53, 28.5)	509.47	4.29 (3.77)	19.51 (14.36)
Basiliximab	3	19.43 (6.25, 60.43)	19.35 (6.21, 60.31)	52.19	4.27 (2.85)	19.34 (7.48)
Sunitinib malate	3	17.57 (5.65, 54.63)	17.51 (5.62, 54.57)	46.67	4.13 (2.71)	17.5 (6.77)
Vincristine	9	17.41 (9.04, 33.53)	17.35 (9.09, 33.13)	138.38	4.11 (3.22)	17.31 (10.00)
Colchicine	4	14.61 (5.47, 39.02)	14.57 (5.47, 38.82)	50.51	3.86 (2.60)	14.56 (6.40)
Evusheld	5	14.29 (5.94, 34.4)	14.25 (5.9, 34.42)	61.53	3.83 (2.67)	14.23 (6.82)
Bisoprolol fumarate	3	13.62 (4.38, 42.33)	13.58 (4.36, 42.33)	34.96	3.76 (2.35)	13.58 (5.26)
Pravastatin	4	12.06 (4.52, 32.2)	12.03 (4.51, 32.05)	40.43	3.59 (2.32)	12.02 (5.29)
Epzicom	3	11.84 (3.81, 36.79)	11.81 (3.79, 36.81)	29.68	3.56 (2.14)	11.80 (4.57)
Atezolizumab	69	11.00 (8.67, 13.96)	10.97 (8.67, 13.88)	615.02	3.43 (3.09)	10.80 (8.85)
Bavencio	3	10.96 (3.53, 34.04)	10.93 (3.51, 34.07)	27.06	3.45 (2.03)	10.93 (4.23)
Rosuvastatin calcium	4	10.83 (4.06, 28.91)	10.81 (4.06, 28.8)	35.58	3.43 (2.17)	10.80 (4.75)
Fludarabine phosphate	19	10.75 (6.85, 16.88)	10.73 (6.84, 16.84)	166.84	3.42 (2.78)	10.68 (7.32)
Rosuvastatin	9	10.51 (5.46, 20.23)	10.49 (5.49, 20.03)	77.10	3.39 (2.49)	10.47 (6.05)
Alemtuzumab	9	10.32 (5.36, 19.87)	10.30 (5.39, 19.67)	75.45	3.36 (2.47)	10.28 (5.94)
Ipilimumab	40	10.29 (7.53, 14.06)	10.27 (7.51, 14.05)	331.50	3.35 (2.90)	10.18 (7.84)
Fingolimod	4	10.12 (3.79, 27.00)	10.10 (3.79, 26.91)	32.76	3.33 (2.07)	10.09 (4.44)
Bortezomib	69	10.00 (7.88, 12.69)	9.98 (7.89, 12.63)	548.19	3.30 (2.96)	9.83 (8.05)
Ribavirin	8	9.98 (4.98, 19.98)	9.96 (5.02, 19.78)	64.35	3.31 (2.37)	9.94 (5.56)
Oxaliplatin	67	9.93 (7.80, 12.64)	9.91 (7.83, 12.54)	528.00	3.29 (2.94)	9.76 (7.98)
Dabrafenib	6	9.89 (4.44, 22.06)	9.87 (4.42, 22.05)	47.79	3.30 (2.23)	9.86 (5.04)
Pembrolizumab	93	9.49 (7.73, 11.66)	9.47 (7.78, 11.52)	689.07	3.21 (2.92)	9.28 (7.81)

ROR, reporting odds ratio; CI, confidence interval; PRR, proportional reporting ratio; *χ*^2^, chi-square; IC, information component; IC-2SD, the lower limit of 95% CI of the IC; EBGM, empirical Bayesian geometric mean; EBGM05, the lower limit of 95% CI of EBGM.

**Table 3:** Summary of algorithms.

Algorithms	Equation	Criteria
ROR	ROR $=\frac{\left(a/c\right)}{\left(b/d\right)}=\frac{ad}{bc}$	a ≥ 3, 95%CI (lower limit) > 1
	95% CI $=e^{\ln\left(ROR\right)\;\pm \;1.96\sqrt{\left(\frac{1}{a}+\frac{1}{b}+\frac{1}{c}+\frac{1}{d}\right)}}$	
PRR	PRR $=\frac{a/(a+b)}{c/(c+d)}$	a ≥ 3, 95%CI (lower limit) > 1, PRR ≥ 2, $\chi^{2}$ ≥ 4
	95% CI $=e^{\ln(PRR)\pm \;1.96\sqrt{\frac{1}{a}-\frac{1}{a+b}+\frac{1}{c}-\frac{1}{c+d}}}$	
	$\chi^{2}=\frac{{\left(ad-bc\right)}^{2}(a+b+c+d)}{\left(a+b)(a+c)(c+d)(b+d\right)}$	
BCPNN	IC $=\log_{2}\frac{p(x,y)}{p\left(x\right)p(y)}=\log_{2}\frac{a(a+b+c+d)}{\left(a+b)(a+c\right)}$	IC-2SD > 0
	E(IC) $=\log_{2}\frac{\left(a+\gamma 11)(a+b+c+d+\alpha )(a+b+c+d+\beta \right)}{\left(a+b+c+d+\gamma )(a+b+\alpha 1)(a+c+\beta 1\right)}$	
	V(IC) $=\frac{1}{{(\ln2)}^{2}}\left\{\left[\frac{\left(a+b+c+d\right)-a+\gamma -\gamma 11}{\left(a+\gamma 11)(1+a+b+c+d+\gamma \right)}\right]+\left[\frac{\left(a+b+c+d\right)-\left(a+b\right)+\alpha -\alpha 1}{\left(a+b+\alpha 1)(1+a+b+c+d+\alpha \right)}\right]+\left[\frac{\left(a+b+c+d\right)-\left(a+c\right)+\beta -\beta 1}{\left(a+c+\beta 1)(1+a+b+c+d+\beta \right)}\right]\right\}$	
	$\gamma =\gamma 11\frac{\left(a+b+c+d+\alpha )(a+b+c+d+\beta \right)}{\left(a+b+\alpha 1)(a+c+\beta 1\right)}$	
	IC-2SD = E(IC) − 2$\sqrt{V(IC)}$	
	p.s. α1=β1=1; α=β=2; γ11=1	
MGPS	EBGM $=\frac{a(a+b+c+d)}{\left(a+c)(a+b\right)}$	EBGM05 > 2
	95%CI $=e^{\ln\left(EBGM\right)\;\pm \;1.96\sqrt{\left(\frac{1}{a}+\frac{1}{b}+\frac{1}{c}+\frac{1}{d}\right)}}$	

ROR, reporting odds ratio; CI, confidence interval; PRR, proportional reporting ratio; BCPNN, Bayesian confidence propagation neural network; *χ*^2^, chi-square; IC, information component; IC-2SD, the lower limit of 95% CI of the IC; MGPS, multi-gene pathway score.

## Data Availability

Publicly available datasets were analyzed in this study. The data can be found here: (https://fs.fda.gov/extensions/FPD-QDE-FAERS/FPD-QDE-FAERS.html) (accessed on 28 September 2024). These datasets can be found at the following URLs: deCODE (https://www.decode.com/summarydata/) (accessed on 10 October 2024); and UKB-PPP (https://www.synapse.org/Synapse:syn51365301) (accessed on 10 October 2024). GBS GWAS data can be accessed from FinnGen (https://www.finngen.fi) (accessed on 10 October 2024) and UK Biobank (https://www.ukbiobank.ac.uk) (accessed on 28 September 2024). Any data generated during the study process are available upon reasonable request by emailing Jianxiong Gui (gui199508046@163.com). The data are not publicly available due to [**Principle of Confidentiality**].

## Supplementary Materials

The following supporting information can be downloaded at: https://www.mdpi.com/article/10.3390/ph18040498/s1, Figure S1: The QQ plot derived from the GWAS meta-analysis; Figure S2: The Manhattan plot showing genetic risk loci significantly associated with GBS identified through genetic analysis. Figure S3: PPI network analyzing interactions between 73 GBS susceptibility proteins and known drug targets; Table S1: The results for drug targets, from the Drugbank database; Table S2: The results of independent significant SNPs from GWAS-meta analysis; Table S3a: The results of MR analysis between plasma protein pQTLs from deCODE and GBS GWAS-mate data, including sensitivity analyses; Table S3b: The results of MR analysis between plasma protein pQTLs from UKB_PPP and GBS GWAS-mate data, including sensitivity analyses. Table S3c: The results of reverse MR analyses, including Decode and UKB_PPP; Table S4: The results of PPI analysis.

## Abbreviations

The following abbreviations are used in this manuscript:

GBS	Guillain–Barré Syndrome
MR	Mendelian Randomization
PPI	Protein–protein Interaction
FAERS	Food and Drug Administration Adverse Event Reporting System
ROR	Reporting Odds Ratio
BCPNN	Bayesian Confidence Propagation Neural Network
MGPS	Multi-item Gamma-Poisson Shrinker
p-QTLs	protein Quantitative Tait Loci
FDR	False Discovery Rate
CI	Confidence Interval
PRR	Proportional Reporting Ratio
TNF	Tumor Necrosis Factor
ICIs	Immune Checkpoint Inhibitors
RRM1	Ribonucleotide Reductase M1

## References

[1] Wijdicks E.F.M., Klein C.J. (2017). Guillain-Barré Syndrome. Mayo Clin. Proc..

[2] Xu L., Zhao C., Bao Y., Liu Y., Liang Y., Wei J., Liu G., Wang J., Zhan S., Wang S. (2024). Variation in worldwide incidence of Guillain-Barré Syndrome: A population-based study in urban China and existing global evidence. Front. Immunol..

[3] Kozyreva A.A., Bembeeva R.T., Druzhinina E.S., Zavadenko N.N. (2023). Guillain-Barre Syndrome in children. Zh. Nevrol. Psikhiatr. Im. S. S. Korsakova.

[4] Haber P., DeStefano F., Angulo F.J., Iskander J., Shadomy S.V., Weintraub E., Chen R.T. (2004). Guillain-Barré Syndrome following influenza vaccination. JAMA.

[5] Awong I.E., Dandurand K.R., Keeys C.A., Maung-Gyi F.A. (1996). Drug-associated Guillain-Barré Syndrome: A literature review. Ann. Pharmacother..

[6] Fagius J., Osterman P.O., Sidén A., Wiholm B.E. (1985). Guillain-Barré Syndrome following zimeldine treatment. J. Neurol. Neurosurg. Psychiatry.

[7] Raschetti R., Maggini M., Popoli P., Caffari B., Da Cas R., Menniti-Ippolito F., Spila-Alegiani S., Traversa G. (1995). Gangliosides and Guillain-Barré Syndrome. J. Clin. Epidemiol..

[8] Okuyan E., Cakar M.A., Dinckal M.H. (2010). Guillain-Barré Syndrome after thrombolysis with streptokinase. Cardiol. Res. Pract..

[9] Gerriets V., Goyal A., Khaddour K. (2025). Tumor Necrosis Factor Inhibitors. StatPearls.

[10] Mikami T., Liaw B., Asada M., Niimura T., Zamami Y., Green-LaRoche D., Pai L., Levy M., Jeyapalan S. (2021). Neuroimmunological adverse events associated with immune checkpoint inhibitor: A retrospective, pharmacovigilance study using FAERS database. J. Neurooncol..

[11] Li Y., Zhang X., Zhao C. (2021). Guillain-Barré Syndrome-Like Polyneuropathy Associated with Immune Checkpoint Inhibitors: A Systematic Review of 33 Cases. BioMed Res. Int..

[12] Fan Q., Hu Y., Wang X., Zhao B. (2021). Guillain-Barré Syndrome in patients treated with immune checkpoint inhibitors. J. Neurol..

[13] Abrahao A., Tenório P.H.d.M., Rodrigues M., Mello M., Nascimento O.J.M. (2024). Guillain-Barré Syndrome and checkpoint inhibitor therapy: Insights from pharmacovigilance data. BMJ Neurol. Open.

[14] Zheng X., Fang Y., Song Y., Liu S., Liu K., Zhu J., Wu X. (2023). Is there a causal nexus between COVID-19 infection, COVID-19 vaccination, and Guillain-Barré Syndrome?. Eur. J. Med. Res..

[15] Malekpour M., Khanmohammadi S., Meybodi M.J.E., Shekouh D., Rahmanian M.R., Kardeh S., Azarpira N. (2023). COVID-19 as a trigger of Guillain-Barré Syndrome: A review of the molecular mechanism. Immun. Inflamm. Dis..

[16] Waheed S., Bayas A., Hindi F., Rizvi Z., Espinosa P.S. (2021). Neurological Complications of COVID-19: Guillain-Barre Syndrome Following Pfizer COVID-19 Vaccine. Cureus.

[17] Braish J.S., Kugler E., Jabbour E., Woodman K., Ravandi F., Nicholas S., Jain N., Kantarjian H., Sasaki K. (2024). Incidence and Clinical Presentation of Severe Neurotoxicity from Nelarabine in Patients with T-Cell Acute Lymphoblastic Leukemia. Clin. Lymphoma Myeloma Leuk..

[18] DeAngelo D.J. (2009). Nelarabine for the treatment of patients with relapsed or refractory T-cell acute lymphoblastic leukemia or lymphoblastic lymphoma. Hematol. Oncol. Clin. N. Am..

[19] Alli N., Lou-Hing A., Bolt E.L., He L. (2024). POLD3 as Controller of Replicative DNA Repair. Int. J. Mol. Sci..

[20] Curik N., Polivkova V., Burda P., Koblihova J., Laznicka A., Kalina T., Kanderova V., Brezinova J., Ransdorfova S., Karasova D. (2021). Somatic Mutations in Oncogenes Are in Chronic Myeloid Leukemia Acquired De Novo via Deregulated Base-Excision Repair and Alternative Non-Homologous End Joining. Front. Oncol..

[21] Mehawej C., Chouery E., Azar-Atallah S., Shebaby W., Delague V., Mansour I., Mustapha M., Lefranc G., Megarbane A. (2023). POLD3 deficiency is associated with severe combined immunodeficiency, neurodevelopmental delay, and hearing impairment. Clin. Immunol. Orlando Fla.

[22] Wu H., Huang X., Chen S., Li S., Feng J., Zouxu X., Xie Z., Xie X., Wang X. (2020). Comprehensive analysis of the NME gene family functions in breast cancer. Transl. Cancer Res..

[23] Chen C.-W., Wang H.-L., Huang C.-W., Huang C.-Y., Lim W.K., Tu I.-C., Koorapati A., Hsieh S.-T., Kan H.-W., Tzeng S.-R. (2019). Two separate functions of NME3 critical for cell survival underlie a neurodegenerative disorder. Proc. Natl. Acad. Sci. USA.

[24] Kamakura K., Kaida K., Kusunoki S., Miyamoto N., Masaki T., Nakamura R., Motoyoshi K., Fukuda J. (2005). Harmful effects of anti-GalNAc-GD1a antibodies and TNF-alpha on rat dorsal root ganglia. J. Peripher. Nerv. Syst. JPNS.

[25] Hartung H.P. (1993). Immune-mediated demyelination. Ann. Neurol..

[26] Nyati K.K., Prasad K.N., Verma A., Paliwal V.K. (2010). Correlation of matrix metalloproteinases-2 and -9 with proinflammatory cytokines in Guillain-Barré Syndrome. J. Neurosci. Res..

[27] Solomon A.J., Spain R.I., Kruer M.C., Bourdette D. (2011). Inflammatory neurological disease in patients treated with tumor necrosis factor alpha inhibitors. Mult. Scler. Houndmills Basingstoke Engl..

[28] Stübgen J.-P. (2008). Tumor necrosis factor-alpha antagonists and neuropathy. Muscle Nerve.

[29] Tristano A.G. (2010). Neurological adverse events associated with anti-tumor necrosis factor α treatment. J. Neurol..

[30] Shitara K., Ajani J.A., Moehler M., Garrido M., Gallardo C., Shen L., Yamaguchi K., Wyrwicz L., Skoczylas T., Bragagnoli A.C. (2022). Nivolumab plus chemotherapy or ipilimumab in gastro-oesophageal cancer. Nature.

[31] Fultang L., Panetti S., Ng M., Collins P., Graef S., Rizkalla N., Booth S., Lenton R., Noyvert B., Shannon-Lowe C. (2019). MDSC targeting with Gemtuzumab ozogamicin restores T cell immunity and immunotherapy against cancers. EBioMedicine.

[32] Horn L., Mansfield A.S., Szczęsna A., Havel L., Krzakowski M., Hochmair M.J., Huemer F., Losonczy G., Johnson M.L., Nishio M. (2018). First-Line Atezolizumab plus Chemotherapy in Extensive-Stage Small-Cell Lung Cancer. N. Engl. J. Med..

[33] von Essen M.R., Chow H.H., Holm Hansen R., Buhelt S., Sellebjerg F. (2023). Immune reconstitution following alemtuzumab therapy is characterized by exhausted T cells, increased regulatory control of proinflammatory T cells and reduced B cell control. Front. Immunol..

[34] Li W., Mei M., Liu T., Zhang S., Wang Z., Suo Y., Wang S., Liu Y., Zhang N., Lu W. (2022). Identification of PDCD1 and PDCD1LG2 as Prognostic Biomarkers and Associated with Immune Infiltration in Hepatocellular Carcinoma. Int. J. Gen. Med..

[35] Esfahani K., Buhlaiga N., Thébault P., Lapointe R., Johnson N.A., Miller W.H. (2019). Alemtuzumab for Immune-Related Myocarditis Due to PD-1 Therapy. N. Engl. J. Med..

[36] Lee S.U., Ahn K.-S., Sung M.H., Park J.-W., Ryu H.W., Lee H.-J., Hong S.-T., Oh S.-R. (2014). Indacaterol inhibits tumor cell invasiveness and MMP-9 expression by suppressing IKK/NF-κB activation. Mol. Cells.

[37] Chang S.-H., Tian X.-B., Wang J., Liu M.-Q., Huang C.-N., Qi Y., Zhang L.-J., Gao C.-L., Zhang D.-Q., Sun L.-S. (2020). Increased Cerebrospinal Fluid Uric Acid Levels in Guillain-Barré Syndrome. Front. Neurol..

[38] Bansil S., Mithen F.A., Singhal B.S., Cook S.D., Rohowsky-Kochan C. (1992). Elevated neopterin levels in Guillain-Barré Syndrome. Further evidence of immune activation. Arch. Neurol..

[39] Benito-León J., Porta-Etessam J. (2001). Guillain-Barré Syndrome and allopurinol-induced hypersensitivity. Eur. Neurol..

[40] Liu X., Liu L., Zhang J. (2024). Causal role of the pyrimidine deoxyribonucleoside degradation superpathway mediation in Guillain-Barré Syndrome via the HVEM on CD4 + and CD8 + T cells. Sci. Rep..

[41] Shimony S., DeAngelo D.J., Luskin M.R. (2024). Nelarabine: When and how to use in the treatment of T-cell acute lymphoblastic leukemia. Blood Adv..

[42] Chun H.G., Leyland-Jones B., Cheson B.D. (1991). Fludarabine phosphate: A synthetic purine antimetabolite with significant activity against lymphoid malignancies. J. Clin. Oncol. Off. J. Am. Soc. Clin. Oncol..

[43] Hess J.R., Greenberg N.A. (2012). The role of nucleotides in the immune and gastrointestinal systems: Potential clinical applications. Nutr. Clin. Pract. Off. Publ. Am. Soc. Parenter. Enter. Nutr..

[44] Rothman K.J., Lanes S., Sacks S.T. (2004). The reporting odds ratio and its advantages over the proportional reporting ratio. Pharmacoepidemiol. Drug Saf..

[45] Evans S.J., Waller P.C., Davis S. (2001). Use of proportional reporting ratios (PRRs) for signal generation from spontaneous adverse drug reaction reports. Pharmacoepidemiol. Drug Saf..

[46] Bate A., Lindquist M., Edwards I.R., Olsson S., Orre R., Lansner A., De Freitas R.M. (1998). A Bayesian neural network method for adverse drug reaction signal generation. Eur. J. Clin. Pharmacol..

[47] Szarfman A., Machado S.G., O’Neill R.T. (2002). Use of screening algorithms and computer systems to efficiently signal higher-than-expected combinations of drugs and events in the US FDA’s spontaneous reports database. Drug Saf..

[48] Ferkingstad E., Sulem P., Atlason B.A., Sveinbjornsson G., Magnusson M.I., Styrmisdottir E.L., Gunnarsdottir K., Helgason A., Oddsson A., Halldorsson B.V. (2021). Large-scale integration of the plasma proteome with genetics and disease. Nat. Genet..

[49] Sun B.B., Chiou J., Traylor M., Benner C., Hsu Y.-H., Richardson T.G., Surendran P., Mahajan A., Robins C., Vasquez-Grinnell S.G. (2023). Plasma proteomic associations with genetics and health in the UK Biobank. Nature.

[50] Turner S.D. (2018). qqman: An R package for visualizing GWAS results using Q-Q and manhattan plots. J. Open Source Softw..

[51] Staley J. jrs95/gassocplot. Published online 17 February 2025. https://github.com/jrs95/gassocplot.

[52] Watanabe K., Taskesen E., van Bochoven A., Posthuma D. (2017). Functional mapping and annotation of genetic associations with FUMA. Nat. Commun..

[53] Bourgault J., Abner E., Manikpurage H.D., Pujol-Gualdo N., Laisk T., Gobeil É., Gagnon E., Girard A., Mitchell P.L., Estonian Biobank Research Team (2023). Proteome-Wide Mendelian Randomization Identifies Causal Links Between Blood Proteins and Acute Pancreatitis. Gastroenterology.

[54] A Practical Guide to Methods Controlling False Discoveries in Computational Biology|Genome Biology|Full Text. https://genomebiology.biomedcentral.com/articles/10.1186/s13059-019-1716-1.

[55] Szklarczyk D., Gable A.L., Nastou K.C., Lyon D., Kirsch R., Pyysalo S., Doncheva N.T., Legeay M., Fang T., Bork P. (2021). The STRING database in 2021: Customizable protein-protein networks, and functional characterization of user-uploaded gene/measurement sets. Nucleic Acids Res..

