# Supplementary material for: Mechanistic Insights into Drug-Induced Guillain–Barré Syndrome: A Large-Cohort Analysis of the FAERS Database
Source: Pharmaceuticals (Basel). 2025 Mar 29;18(4):498. doi: 10.3390/ph18040498 (PMC12030582; doi:10.3390/ph18040498)
Supplement: Supplementary file 1 [file pharmaceuticals-18-00498-s001.zip › Supplementary Figures.pdf]

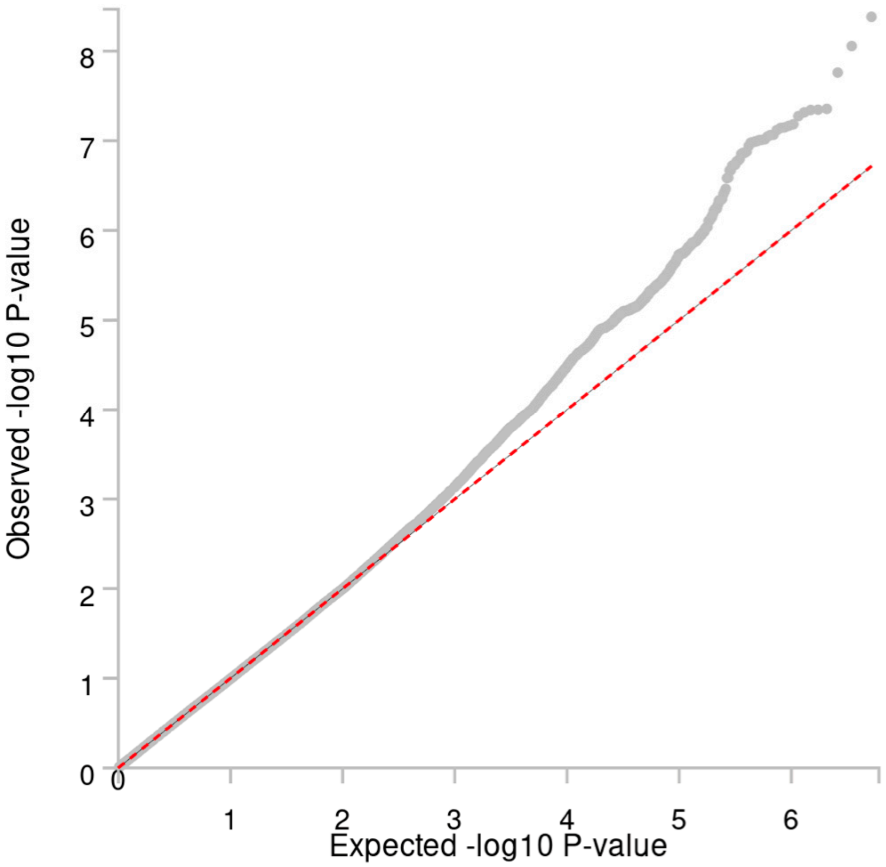

Figure S1. The QQ plot derived from the GWAS meta-analysis.

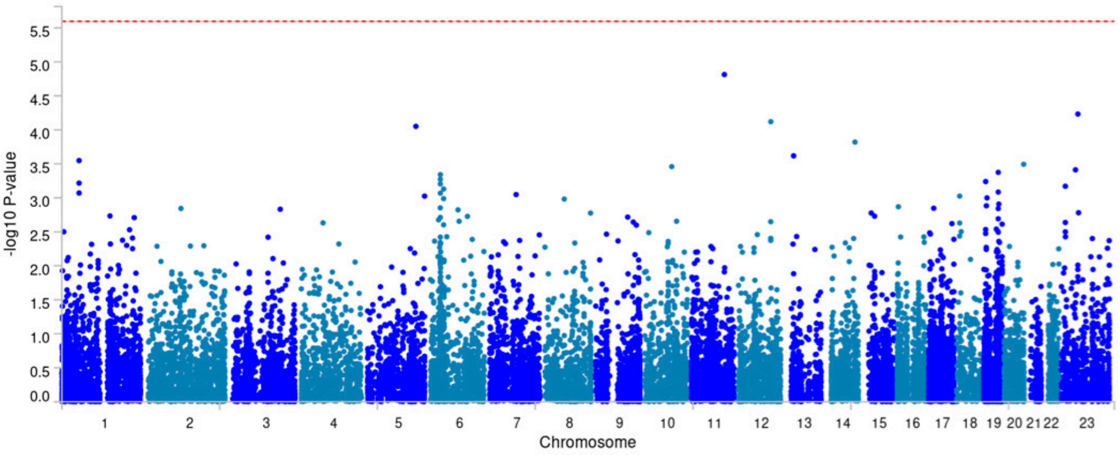

Figure S2. Manhattan plot showing genetic risk loci significantly associated with GBS identified through genetic analysis.

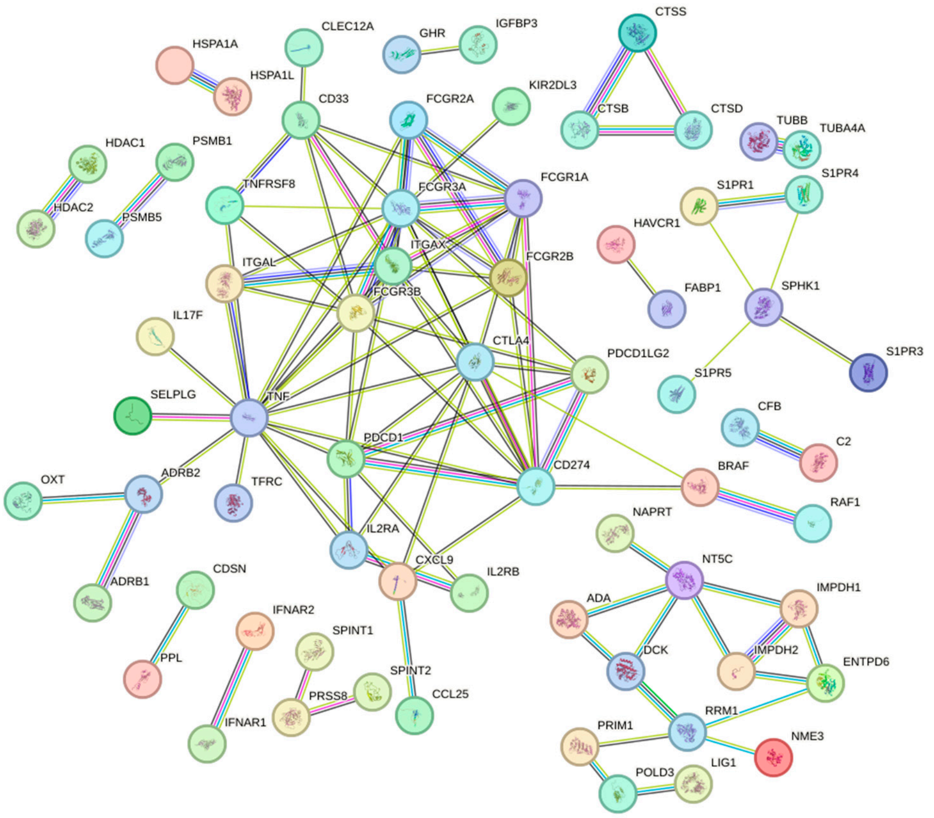

Figure S3. PPI network analyzing interactions between 73 GBS susceptibility proteins and known drug targets.
